# Up-to-Date Clinical Practice in Percutaneous Coronary Intervention for Multivessel Disease∗

**DOI:** 10.1016/j.jacasi.2023.01.001

**Published:** 2023-03-11

**Authors:** Sung-Jin Hong

**Affiliations:** Severance Hospital, Yonsei University College of Medicine, Seoul, South Korea

**Keywords:** coronary stent, intravascular ultrasound, percutaneous coronary intervention

**Figure:**
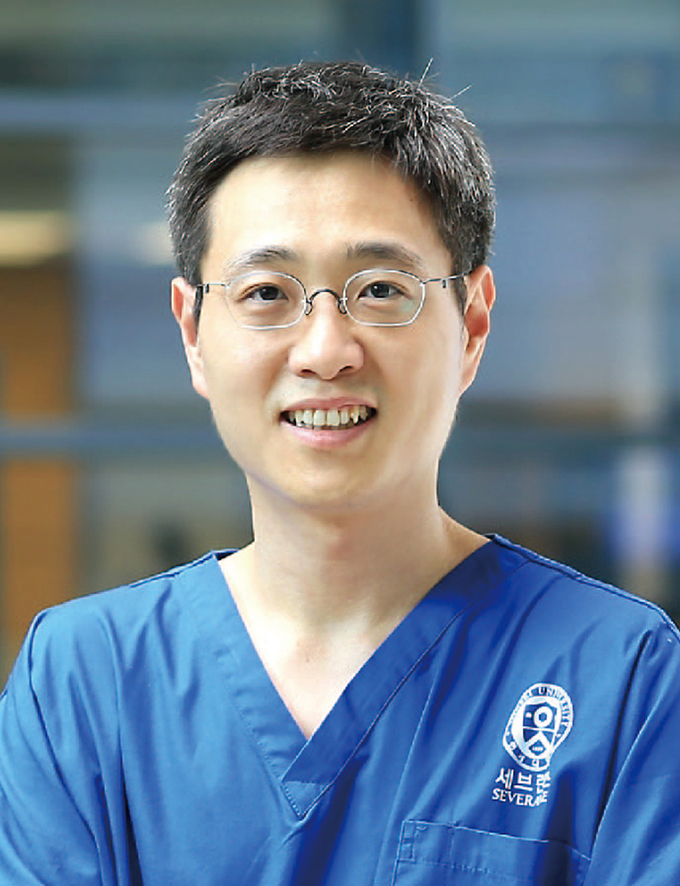

Percutaneous coronary intervention (PCI) for multivessel disease in coronary artery disease is challenging despite recent advances in devices and techniques, particularly for lesions involving complex coronary anatomy. Coronary artery bypass graft (CABG) is the preferred revascularization strategy for this subset of patients.^1^^,^^2^ In addition, although there is evidence of improved long-term outcomes with use of intravascular imaging such as intravascular ultrasound (IVUS),^3^ its use in the PCI arm among several randomized trials comparing clinical efficacy of revascularization strategies of PCI VS CABG was relatively low or not reported in patients with multivessel disease. In this issue of *JACC: Asia*, Yamamoto et al^4^ evaluated clinical outcomes after optimal IVUS-guided PCI in patients undergoing multivessel PCI from the OPTIVUS-Complex PCI (Optimal Intravascular Ultrasound Guided Complex Percutaneous Coronary Intervention) multivessel cohort. This prospective, multicenter, single-arm cohort enrolled 1,021 patients undergoing multivessel PCI, including left anterior descending coronary artery; the goal of using IVUS was to meet the prespecified criteria for optimal stent expansion. The primary endpoint was major adverse cardiac and cerebrovascular events, a composite of death, myocardial infarction, stroke, and any coronary revascularization. When occurrence of the primary endpoint was compared with the predefined performance goals derived from a previous registry, the cumulative 1-year incidence of the primary endpoint was 10.3% (95% CI: 8.4%-12.2%), which was significantly lower than the predefined PCI performance goal of 27.5%.

The investigators should be congratulated for demonstrating the superiority of the predefined PCI performance goal of multivessel PCI in a setting of up-to-date clinical practice.^4^ It is notable that all patients enrolled in this registry underwent PCI under IVUS guidance. More importantly, IVUS-guided PCI was performed by using the prespecified optimization criteria for stent expansion: minimum stent area greater than the distal reference lumen area when stent length was ≥28 mm and minimal stent area >80% by average reference lumen area when stent length was <28 mm. Even though the achievement of these predefined optimization criteria was not associated with improvement in outcome, it may be attributed to relatively less complex lesions despite treatment of multiple lesions or need of other optimization criteria for this subset of the patients or lesions. In addition to IVUS usage for all PCIs, physiological selection of PCI, radial approach, use of contemporary generation stents, or refraining from routine follow-up coronary angiography also add evidence for contemporary PCI for multivessel disease. This cohort also reflects well the contemporary strategies for medical therapy after PCI such as shorter dual-antiplatelet therapy followed by use of P2Y_12_ inhibitors as a monotherapy and high-intensity statin therapy.^5^ Management of risk factors, including medical therapy for secondary prevention, may be important, especially for patients with multivessel disease.^6^

The following issues also need to be considered for interpretation of the major findings of this study.^4^ Although the outcomes of PCI in the OPTIVUS-Complex PCI study were associated with a significantly lower rate of the primary endpoint than was the predefined PCI performance, the performance goal of PCI was based on patients treated between 2005 and 2007. A relatively larger proportion of low SYNTAX (Synergy Between Percutaneous Coronary Intervention With Taxus and Cardiac Surgery) score (<23) (79%) and a smaller proportion of patients with 3-vessel disease (20%) in this study also need to be considered. In this analysis, the investigators only reported the 1-year outcomes relative to the predefined PCI performance; however, long-term outcomes assessing noninferiority relative to the performance goal at 5 years can provide evidence of multivessel PCI, particularly with IVUS usage. Although the investigators did not focus on further analyses of IVUS parameters, derivation and validation of various optimization criteria associated with better clinical outcomes using the OPTIVUS-Complex PCI study cohort also can be possible in the future. Several ongoing randomized trials evaluating the benefit of intravascular imaging guidance, particularly for complex lesions such as left main, bifurcation lesions requiring 2 stents, or chronic total occlusions, may provide the values of intravascular imaging–guided stent optimization: the IMPROVE trial (Impact on Revascularization Outcomes of Intravascular Ultrasound-Guided Treatment of Complex Lesions and Economic Impact; NCT04221815), the IVUS-CHIP trial (Intravascular Ultrasound Guidance for Complex High-Risk Indicated Procedures; NCT04854070), the OPTIMAL trial (OPtimizaTIon of Left MAin PCI With IntravascuLar Ultrasound; NCT04111770), the DKCRUSH VIII trial (IVUS-Guided DK Crush Stenting Technique for Patients With Complex Bifurcation Lesions; NCT03770650), the ILUMIEN IV trial (Optical Coherence Tomography [OCT] Guided Coronary Stent Implantation Compared to Angiography: a Multicenter Randomized Trial in PCI; NCT03507777), the OCCUPI trial (Optical Coherence Tomography-Guided Coronary Intervention in Patients With Complex Lesions: a Randomized Controlled Trial; NCT03625908), the RENOVATE trial (Intravascular Imaging- Versus Angiography-Guided Percutaneous Coronary Intervention for Complex Coronary Artery Disease; NCT03381872), and the OCTIVUS trial (Optical Coherence Tomography Versus Intravascular Ultrasound Guided Percutaneous Coronary Intervention; NCT03394079).

In summary, Yamamoto et al^4^ provide valuable evidence regarding up-to-date clinical practice with PCI for multivessel disease from the OPTIVUS-Complex PCI study multivessel cohort. The study performed multivessel PCI using IVUS guidance with predefined optimization criteria in the setting of a contemporary clinical practice such as use of new-generation drug-eluting stents, greater use of radial approaches, use of high-intensity statins, and relatively shorter dual-antiplatelet therapy followed by P2Y_12_ inhibitor monotherapy.

## Funding Support and Author Disclosures

The author has reported that he has no relationships relevant to the contents of this paper to disclose.
